# Microbial living materials promote coral larval settlement

**DOI:** 10.1093/pnasnexus/pgaf268

**Published:** 2025-09-09

**Authors:** Natalie Levy, Samapti Kundu, Marnie Freckelton, Julie Dinasquet, Isabel Flores, Claudia T Galindo-Martínez, Martin Tresguerres, Vanessa De La Garza, Yazhi Sun, Zahra Karimi, Crawford Drury, Christopher P Jury, Joshua R Hancock, Shaochen Chen, Michael G Hadfield, R3D Consortium, Ben Jones, Josh Levy, Sean Mahaffey, Aricia Argyris, Mark Aruda, Ian Robertson, Zhenhua Huang, Ayrton Medina-Rodriguez, Mert Gokdepe, Brady Halvorson, Jon Chase, Charlotte White, Cami Dillon, Kristian McDonald, Anna Mikkelsen, Josh Madin, Mollie Asbury, Jessica Reichert, Hendrikje Jorissen, Nina Schiettekatte, Marion Chapeau, Rob Toonen, Chris R Suchocki, Van Wishingrad, Christopher P Jury, Dan Schar, Madeleine Hardt, Claire Lewis, Claire Bardin, Joshua Kualani, Crawford Drury, Kira Hughes, Josh Hancock, Carlo Caruso, Andrea Grottoli, Shannon Dixon, Ann Marie Hulver, Joshua D Voss, Allison Klein, Siddhartha Verma, Alejandro Alvaro, Richard Argall, Kevin Chun, William Hicks, Alex LeBon, John Yeh, Aaron Thode, Océane Boulais, Daniel Wangpraseurt, Samapti Kundu, Natalie Levy, Stefan Kolle, Lindsey Badder, Daniel Wangpraseurt

**Affiliations:** Scripps Institution of Oceanography, University of California San Diego, 8622 Kennel Way, La Jolla, CA 92037, USA; Department of Chemical and NanoEngineering, University of California San Diego, 3291 Voigt Dr, La Jolla, CA 92093, USA; Scripps Institution of Oceanography, University of California San Diego, 8622 Kennel Way, La Jolla, CA 92037, USA; Department of Chemical and NanoEngineering, University of California San Diego, 3291 Voigt Dr, La Jolla, CA 92093, USA; Kewalo Marine Laboratory, University of Hawai’i, 41 Ahui St, Honolulu, HI 96813, USA; Scripps Institution of Oceanography, University of California San Diego, 8622 Kennel Way, La Jolla, CA 92037, USA; Scripps Institution of Oceanography, University of California San Diego, 8622 Kennel Way, La Jolla, CA 92037, USA; Department of Chemical and NanoEngineering, University of California San Diego, 3291 Voigt Dr, La Jolla, CA 92093, USA; Scripps Institution of Oceanography, University of California San Diego, 8622 Kennel Way, La Jolla, CA 92037, USA; Scripps Institution of Oceanography, University of California San Diego, 8622 Kennel Way, La Jolla, CA 92037, USA; Department of Chemical and NanoEngineering, University of California San Diego, 3291 Voigt Dr, La Jolla, CA 92093, USA; Department of Chemical and NanoEngineering, University of California San Diego, 3291 Voigt Dr, La Jolla, CA 92093, USA; Department of Chemical and NanoEngineering, University of California San Diego, 3291 Voigt Dr, La Jolla, CA 92093, USA; Hawai’i Institute of Marine Biology, University of Hawai’i, 46-007 Lilipuna Rd, Kāne'ohe, HI 96744, USA; Hawai’i Institute of Marine Biology, University of Hawai’i, 46-007 Lilipuna Rd, Kāne'ohe, HI 96744, USA; Hawai’i Institute of Marine Biology, University of Hawai’i, 46-007 Lilipuna Rd, Kāne'ohe, HI 96744, USA; Department of Chemical and NanoEngineering, University of California San Diego, 3291 Voigt Dr, La Jolla, CA 92093, USA; Kewalo Marine Laboratory, University of Hawai’i, 41 Ahui St, Honolulu, HI 96813, USA; Scripps Institution of Oceanography, University of California San Diego, 8622 Kennel Way, La Jolla, CA 92037, USA; Department of Chemical and NanoEngineering, University of California San Diego, 3291 Voigt Dr, La Jolla, CA 92093, USA

**Keywords:** bacteria, bioprinting, ecosystem engineering, coral recruitment, coral reef restoration

## Abstract

The global decline of coral reefs calls for new strategies to rapidly restock coral populations and maintain ecosystem functions and services. Low recruitment success on degraded reefs hampers coral sexual propagation and leads to reduced genetic diversity and impaired reef resilience. Here, we introduce a Bacterial Reef Ink (Brink) to assist in coral larval settlement. Brink is a photopolymerized living material that can be rapidly applied to restoration substrates and has been formulated to cultivate two settlement-inducing bacterial strains (*Cellulophaga lytica* and *Thalassotalea euphylliae*). Settlement assays performed with broadcast spawning (*Montipora capitata*) and brooding (*Pocillopora acuta*) Indo-Pacific corals showed that Brink-coated substrates increased settlement >5-fold compared with uncoated control substrates. Brink can be applied as a flat coating or patterned using light-assisted 3D bioprinting, enabling diverse applications in reef restoration and engineering. This approach demonstrates the potential of functional living materials to enhance coral ecosystem engineering and support coral reef rehabilitation.

Significance Statement Coral reefs are rapidly declining due to environmental stress and habitat degradation, with low coral larval settlement posing a major challenge to reef recovery. Here, we introduce a Bacterial Reef Ink (Brink) that successfully cultivates settlement-inducing bacteria in a 3D hydrogel network. Brink can be rapidly applied to restoration substrates via photopolymerization, forming a functional coating that significantly enhances coral settlement compared with uncoated controls. This adaptable approach expands the potential applications of bioengineered materials for coral reef restoration.

## Introduction

Against the backdrop of the mounting pressures that global coral reefs face, such as rising ocean temperatures (1), ocean acidification (2), and pollution (3), replenishment of coral recruitment is vital to ensure their continued persistence. Maintaining coral reef biodiversity and building the reef habitat relies on the successful recruitment of new corals to the reef (4, 5). Efforts in coral restoration typically involve either fragmenting adult corals or cultivating juvenile corals through sexual reproduction. Notably, the latter approach presents the advantage of bolstering genetic diversity (6), which is indispensable for enhancing ecosystem resilience (7). However, the progress of enhancing sexually propagated corals in their natural habitat has been hindered by low settlement rates (8). Therefore, there is an increasing interest in finding innovative ways to enhance coral recruitment in nature (9).

Coral recruitment relies on the sensory physiology of their larvae, which must detect and transduce chemical cues that stimulate settlement and metamorphosis (10, 11). Some of the most well-documented attractants of invertebrate and coral larvae are chemical cues from marine bacterial biofilms and crustose coralline algae (CCA) (8, 12–18), an abundantly distributed functional group of algae that encrust the reef surface (19, 20). More recently, individual strains of gram-negative bacteria derived from biofilms, such as several strains of *Pseudoalteromonas* sp. (12, 13, 21, 22) and *Cellulophaga lytica* (23, 24), have been identified as invertebrate larvae morphogens (12, 13, 21, 22). Bacteria from these genera produce conserved macromolecules, such as lipopolysaccharides (LPSs) (24, 25), that have been found to induce the settlement of invertebrate larvae, including marine worms, *Hydroides elegans* (24), and corals (26, 27). The mechanism by which this was deduced in *H. elegans* larvae was by experimenting with LPS-producing bacterial strains to discover that polysaccharides (O-antigen) were the inductive component (25). The progress in understanding coral settlement induction opens avenues for interventions to enhance recruitment rates on reefs.

Using settlement-inducing bacteria to improve coral recruitment could be a viable strategy for reef restoration on natural, artificial, or hybrid reefs (i.e. man-made structures with biological interventions). Recent advances in biomanufacturing and 3D bioprinting have led to the development of functional living materials that facilitate the controlled growth of cells (28) and microorganisms (29). 3D bioprinting has revolutionized tissue engineering, precision medicine, and therapeutics (30). Recently, such techniques have been further developed for applications in environmental and marine sciences (31), including the engineering of biomimetic materials releasing the chemical cues of CCA (32), microorganism-powered materials (33, 34) that mimic coral tissues (33) and symbiosis (29) as well as functional synthetic biofilms (35–37). Controlling the microenvironment in which bacteria grow through the engineering of living materials may lead to viable ways to enhance coral larval recruitment on natural and hybrid coral reefs.

**Fig. 1:**
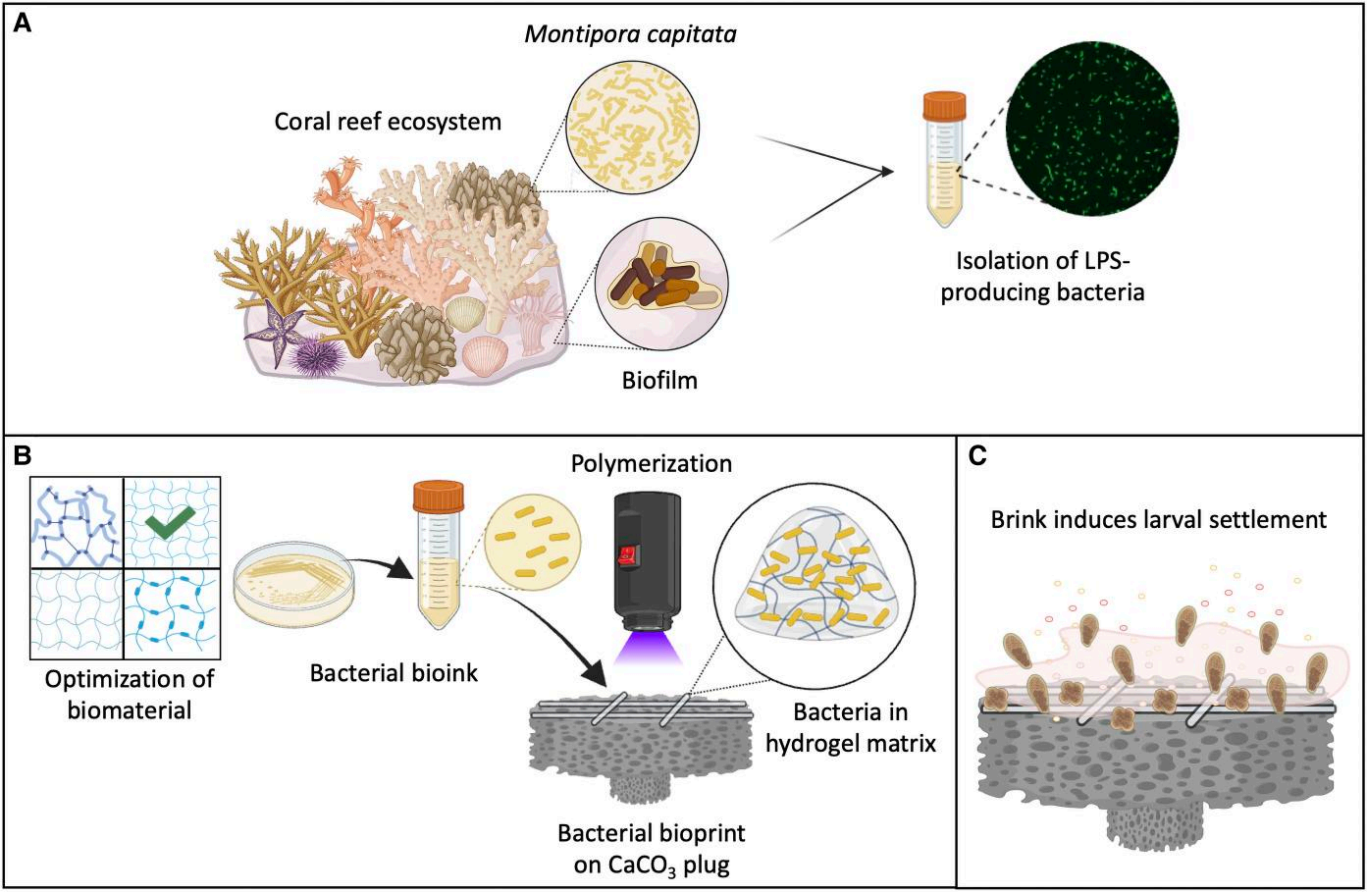
Schematic representation of Brink as a functional living coating designed to enhance coral settlement. A) Harvest and isolation of LPS-producing bacteria from the tissues of the coral *M. capitata* (*T. euphylliae* H1) and marine biofilm (*C. lytica* HI1). B) Optimization of biopolymer mixture for microbial cell viability and mechanical properties. Rapid light-assisted crosslinking of Brink on calcium carbonate (CaCO_3_) plugs, a common restoration material. C) Brink sustains living bacteria that act as biofactories, producing LPS to attract coral larvae from the surrounding environment.

In the present study, we developed a Bacterial Reef Ink (Brink) that enables the biofabrication of hydrogels with settlement-inducing bacteria (Fig. 1). To develop Brink for rapid coating of reef substrates, we first identified suitable settlement-inducing bacteria. The bacterial strains, *Thalassotalea euphylliae* isolated from the coral tissues of *Montipora capitata* (26, 38) and *C. lytica* isolated from marine biofilms (39, 40), were selected based on their previously discovered ability to produce LPS and induce settlement of invertebrate larvae, including corals (23–27, 39). This study highlights the potential of bioengineered living materials for coral rehabilitation and reef restoration (31, 41).

## Results

### Evaluating Brink as a functional microhabitat for bacteria

**Fig. 2:**
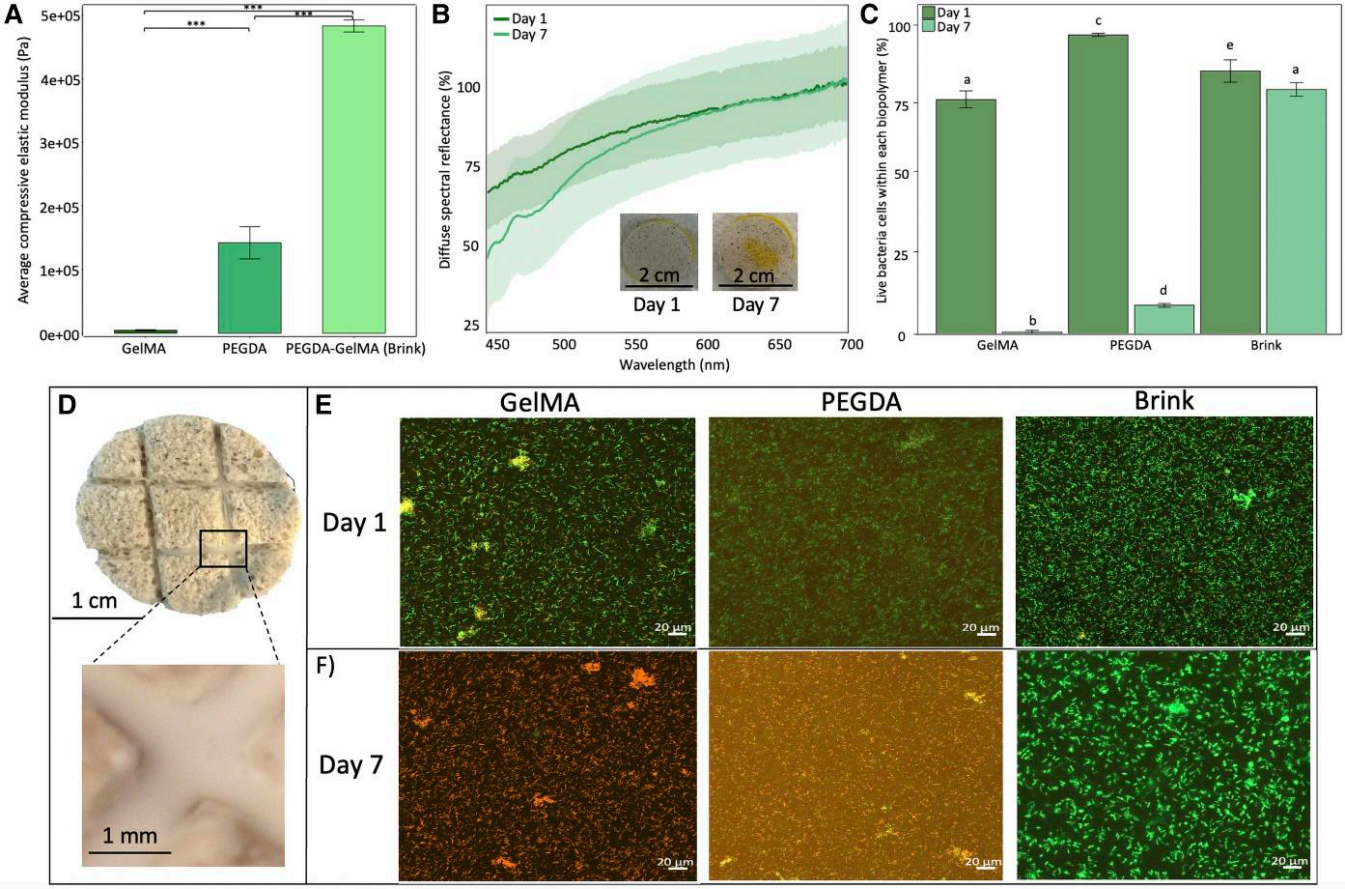
Material properties and bacterial viability in Brink hydrogels. A) Average compressive elastic modulus ± SD (*n* = 3) of GelMA PEGDA, and PEGDA–GelMA (Brink) hydrogels. Significance equivalent to ***P < 0.0001. B) Diffuse spectral reflectance (%) of Brink hydrogels measured on day 1 and day 7. Shaded regions represent 95% CIs (*n* = 5 hydrogels). Insets show the visual change in hydrogel transparency over 7 days. C) Mean percentage of live cells ±SD at day 1 and day 7 (*n* = 3 hydrogels). Letters indicate significance among groups, with the same letters indicating no significance. D) Overview of CaCO_3_ plug with Brink hydrogel in crevices. E) Cell viability of *C. lytica* encapsulated in GelMA, PEGDA, and Brink hydrogels on day 1. Maximum z-projection images (orthogonal view, step size = 20 µm; stack volume 30 mm), showing live and dead cells, in green and red respectively, for GelMA, PEGDA, and Brink hydrogels on day 1. F) Live and dead cells (orthogonal view, step size = 20 µm; stack volume 30 mm) for GelMA, PEGDA, and Brink hydrogels on day 7 after 1 week.

To provide a tunable 3D cell culture environment for the two bacterial strains, we optimized biopolymers based on cell viability, biocompatibility, and hydrogel stability (Fig. 2). Brink hydrogels were characterized by a high Young's modulus (4.83 × 10^5^ ± 9.46 × 10^3^ Pa), which is about 3.4-fold higher than those that only contain poly(ethylene glycol) diacrylate (PEGDA) (1.42 × 10^5^ ± 2.5 × 10^4^ Pa; ANOVA, *P* < 0.0001, Fig. 2B) and >100-fold higher than hydrogels that only contain gelatin methacrylate (GelMA; 4.39 × 10^3^ ± 9.24 × 10^2^ Pa).

Diffuse reflectance (*R*_d_) spectroscopy was used as a noninvasive tool to monitor cell growth and estimate bacterial density of *C. lytica* in GelMA, PEGDA, and Brink hydrogels. The dense bacterial growth was visually apparent according to the yellowing of the initially transparent Brink hydrogel (Fig. 2B, insets) and a reduction in *R*_d_ (450–600 nm) from day 1 to day 7 (42, 43). Confocal imaging suggested that the proportion of dead cells was <12.2% (±10.7 SD) at day 1 for all treatments, and this includes any dead cells before photopolymerization (Fig. 2C). Thus, the short UV exposure (<45 s) and low UV intensity during photo-crosslinking had negligible impact on cell viability. After 7 days of cultivation in hydrogels, Brink hydrogels sustained high cell viability of >81% (±2.23 SD; *P* < 0.0001, Fig. 2D–F). In contrast, cell viability was significantly lower in GelMA hydrogels (0.65% ± 0.54 SD, ∼125-fold lower) and PEGDA hydrogels (9.5% ± 0.64 SD, ∼8.5-fold lower, Fig. 2D–F).

### Settlement-inducing effects of Brink on coral larvae

**Fig. 3:**
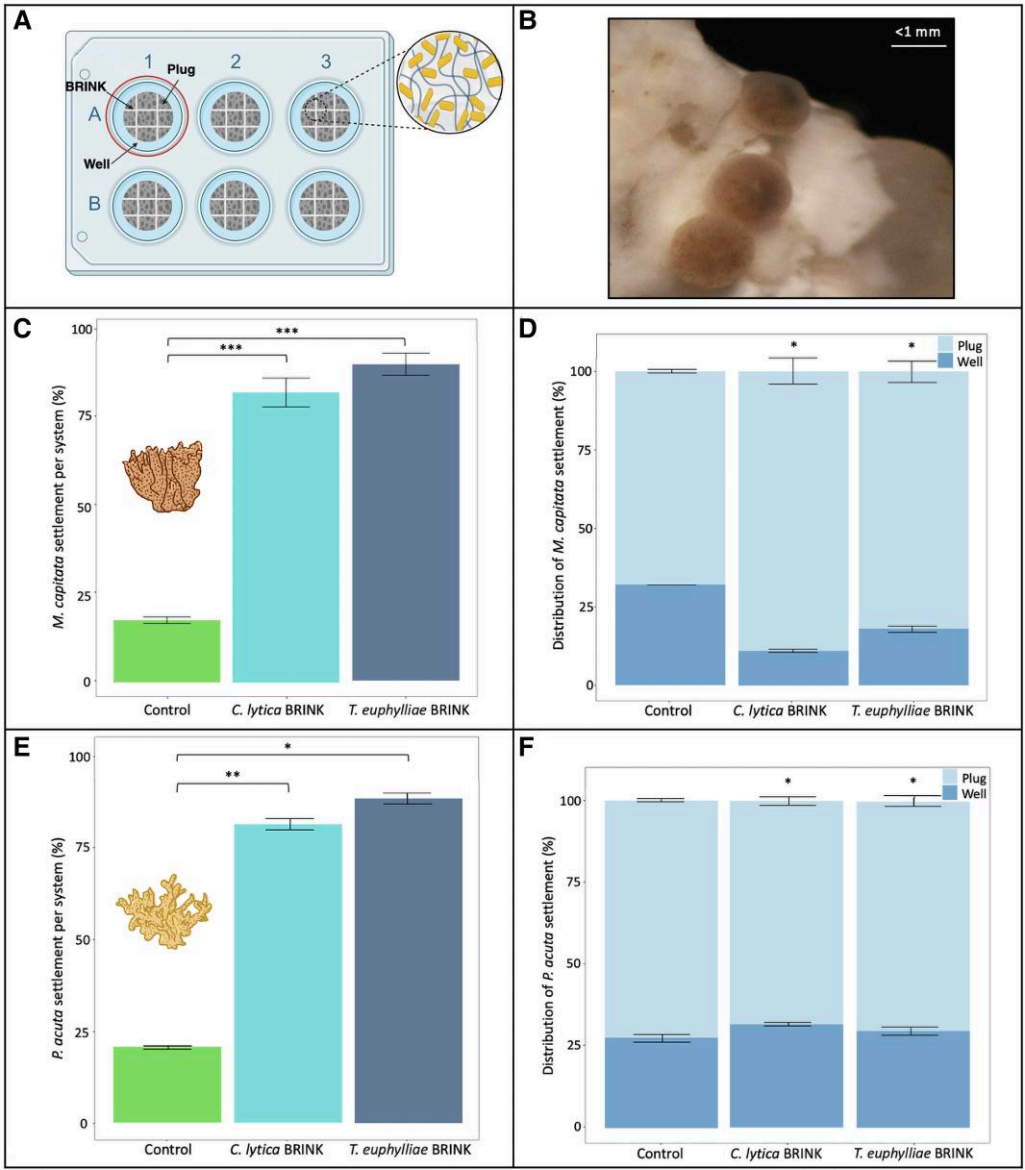
Coral larval settlement response to Brink-coated substrates. A) Experimental setup of settlement plugs in 6-well plates. Settlement was evaluated on the surface of the plug (Brink coated or uncoated) as well as the surrounding surface of each well. B) Example image of settled and attached *M. capitata* larvae on a Brink-coated crevice. C and D) *Montipora capitata* settlement response. C) Total settlement (plug + well) in % of total larvae added (means ± SD, *n* = 6 wells). D) Distribution of settled larvae on the surface of the plug and the surrounding well plate (means ± SD*, n* = 6 wells or plugs). E and F) *Pocillopora acuta* settlement response. E) Total settlement (plug + well) in % of total larvae added (means ± SD, *n* = 6 wells). F) Distribution of settled larvae on the surface of the plug and the surrounding well plate (means ± SD*, n* = 6 wells or plugs). Significance levels are equivalent to *P < 0.05, **P < 0.001, and ***P < 0.0001 (see Figs. S1–S4).

We tested the effect of Brink coatings on larval settlement using the broadcast spawning coral, *M. capitata* and the brooding coral, *Pocillopora acuta* (Fig. 3A–F). Settlement of *M. capitata* (96 h postfertilization; 12–15 h after introduction to assays), was enhanced >5-fold on Brink-coated substrates compared with uncoated controls (*P* < 0.0001, Fig. 3C). There was no significant difference in *M. capitata* settlement rates between *C. lytica* and *T. euphylliae* Brink-coated substrates (Fig. 3C). Over 82% of larvae settled either on the plug or the surface of the well in Brink treatments (Fig. 3D). Over 80% of settled *M. capitata* larvae settled directly on the plug and <20% settled on the surface of the well, regardless of bacterial treatment. The proportion of larvae that settled on the plug increased for Brink-coated substrates (80% ± 2.96 SD and 88% ± 3.668 SD; *P* < 0.05) compared with uncoated controls (70% ± 3.85 SD; Figs. 3D, S1, and S2).

*Pocillopora acuta* larval settlement was enhanced more than 4-fold on Brink-coatings compared with uncoated controls (*T. euphylliae* 88.3% ± 1.50 SD, *P* < 0.05; *C. lytica* 81% ± 1.46 SD, *P* < 0.001; uncoated control 20.6% ± 0.48 SD; Figs. 3E and S3). Similar to *M. capitata* larvae, there was no significant difference in *P. acuta* settlement rates between *C. lytica* and *T. euphylliae* Brink-coated substrates (Fig. 3E). Approximately 70% of the tested larvae preferred to settle directly on the Brink-coated plugs, while around 30% of larvae settled in the surrounding well (*P* < 0.05; Figs. 3F and S4). In contrast, only ∼13% (±0.38 SD) of larvae settled on the plug of the uncoated substrates compared with the surrounding well (Fig. 3F). These results suggest that the larvae settled in response to close proximal contact with the Brink coatings.

### Brink microbial community dynamics in seawater conditions

**Fig. 4:**
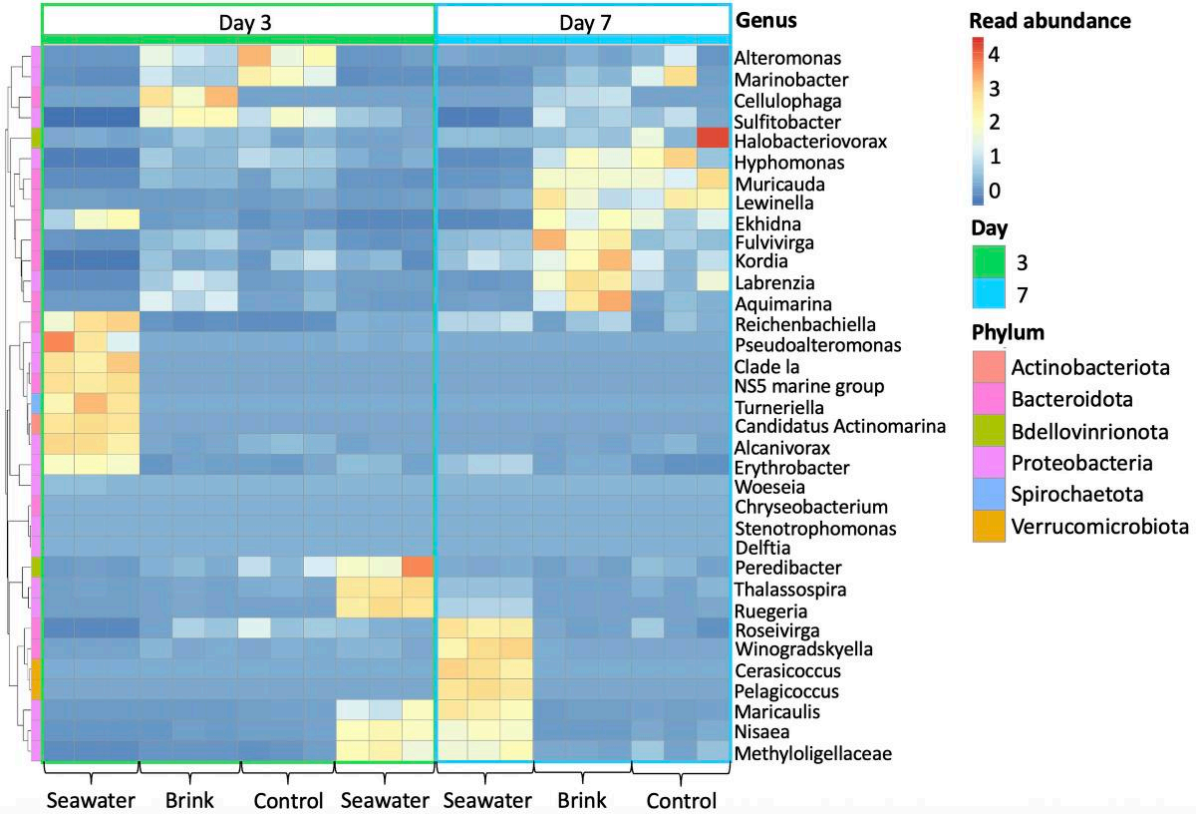
Normalized read abundances of the top 35 bacterial genera from seawater, Brink-coated plugs, and control hydrogel plugs, which were sampled 3 and 7 days post-encapsulation (*n* = 3 per treatment). The first set of seawater replicates represents day 3 seawater, while the second set represents seawater after the day 3 water change. Scale bars and cell colors indicate the distance from the raw score to the mean standard deviation, with genera clustered by similarity.

To assess the suitability of Brink for in situ applications, we tested the bacterial community stability of Brink hydrogels, using *C. lytica* as our model strain, in natural seawater for 1 week (Fig. 4). On day 3, *Cellulophaga* was the most abundant genus associated with Brink-coated plugs, indicating successful growth within the hydrogel matrix, despite the presence of opportunistic bacteria in the surrounding seawater (Figs. 4 and S5). Notably, *C. lytica* was not observed outside the hydrogel, thus suggesting effective immobilization with the hydrogel matrix. Reads from the genus *Cellulophaga* in surrounding seawater were near or below detection thresholds, appearing in only two samples on day 3. They were absent after the water change on day 3 and not detected beyond day 7. This suggests that these were transient, background strains naturally present in the seawater (44), and not *C. lytica* from the hydrogel matrix. In contrast, the surrounding seawater was dominated by different bacterial genera over time, with eight genera prevailing on day 3 and another 10 genera persisting after a water change through day 7 (Fig. 4). A similar bacterial community was found associated with Brink-coated and control hydrogels, likely bacteria present in seawater that had an affinity for the hydrogel. By day 7, *Cellulophaga* abundance slightly declined, likely due to initial rapid growth, followed by nutrient depletion (Fig. 4).

## Discussion

We developed a viable microbial living material optimized to cultivate two strains of coral larval settlement-inducing bacteria. To ensure stability and bacterial colonization, we employed two well-established photocrosslinkable polymers: PEGDA and GelMA (33). PEGDA, a synthetic polymer, provides mechanical stiffness and antifouling properties (45), while GelMA, a natural biopolymer, enhances biocompatibility and biodegradability with tunable mechanical properties (45, 46). The combination of PEGDA and GelMA creates a structurally robust yet porous hydrogel matrix, promoting high bacterial cell viability and sustained growth over seven days, outperforming other tested polymers (Fig. 2 (47, 48)). A slight decline in *C. lytica* under seawater conditions suggests that optimizing bacterial loading densities may further improve stability.

PEGDA–GelMA hydrogels have been widely used for various living cell applications. For example, PEGDA–GelMA hybrid scaffolds have been employed to replicate complex microenvironments, including those of malignant melanoma cells, due to their mechanical strength, degradation resistance, and ability to sustain high cell viability (49). These hydrogels are commonly optimized for enhanced mechanical integrity, biodegradability, and swelling properties, as observed in this study (50). Collectively, our findings highlight the versatility of PEGDA–GelMA hydrogels as engineered microenvironments for coral settlement–inducing bacteria.

Settlement assays revealed that Brink successfully enhanced coral larval settlement compared with uncoated controls (Fig. 3A–F). Additionally, larvae preferentially settled on or near Brink coatings (Fig. 3B, D, and F), highlighting the importance of small-scale microhabitat properties in settlement induction. While coral species-specific responses exist (26, 27), the two bacterial strains tested here were found to induce settlement and metamorphosis for various marine invertebrates (23–27, 39, 51). Recent studies highlight the role of microbial byproducts, including tetrabromopyrrole, chemical extracts of CCA, and their associated biofilm communities, in driving coral larval metamorphosis (15, 32, 52). Recently, Freckelton et al. (25) elucidated the function of LPS, specifically the O-antigen polysaccharide component, in stimulating settlement and metamorphosis in *H. elegans* larvae. Their findings demonstrate that LPS from *C. lytica*, *T. euphylliae*, and other inductive wild-type biofilms consistently induce metamorphosis, while LPS from noninductive bacteria does not (25).

Although Brink incorporates monospecific biofilms, it induced a relatively high metamorphosis rate (80–88%) comparable with those reported for natural biofilms or biofilms derived from CCA (26). Sneed et al. (53) also demonstrated that the monospecific biofilm, *Pseudoalteromonas* sp. PS5 induced settlement in two Caribbean coral species at rates similar to or in some cases higher than CCA. Similarly, Tran and Hadfield (26) showed that *T. euphylliae* (strain M23b) strongly induced metamorphosis of *P. damicornis* larvae at 60%, compared with 70% induction by a wild, multispecies biofilm. Brink can offer practical advantages as a rapidly deployable, tunable biofilm, even as a monoculture, avoiding the need for weeks of biofilm development, but with future potential to incorporate co- or poly-culture biofilms to enhance settlement further.

In this study, we focused on developing a living material that enhances settlement, rather than investigating the mechanistic pathways of metamorphosis. Future research is needed to elucidate the long-term interactions between coral larvae, postsettlement survival, Brink hydrogels, associated microbial communities, and interactions with other species that may impact recruitment and survivorship of coral larvae, enabling precise tuning of biomaterial and microbial properties for targeted coral reef restoration applications.

### Applications for coral reef restoration

While microbial 3D bioprinting has shown immense potential in tissue engineering, bioprocess engineering, and biomedicine (31), this study demonstrates that it can be used to control local biochemical landscapes in coral reef ecosystems by creating a living microbial material that enhances coral larval settlement. This finding establishes a previously unexplored application of microbial 3D bioprinting and highlights avenues for utilizing Brink and other living materials in coral reef restoration (31, 41). Brink introduces a scalable, bioengineered material that enhances coral settlement by rapidly delivering settlement-inducing bacteria to coat restoration substrates. Unlike traditional biofilm conditioning methods, Brink allows for immediate application via photopolymerization, eliminating the need for prolonged seawater exposure. Our results demonstrated successful settlement enhancement in both a broadcast spawning and a brooding coral species, highlighting the versatility of Brink as a short-term, sustainable material, which can be used for promoting larval settlement during annual mass spawning events and continuous larval release throughout the year.

As Brink utilizes native Hawaiian bacterial strains targeting local coral settlement and is designed as a sustainable tool for use during key reproductive windows, its natural degradation or eventual fouling minimizes long-term ecological risk to the surrounding environment. Recent work demonstrates the long-term viability of the same PEGDA–GelMA polymer formulation (used in Brink), under natural seawater conditions, indicating its potential robustness in the field (32). While Brink is not inherently antifouling, future versions could integrate surface modifications, such as SLIPS (slippery-liquid infused porous surface) coatings (54), which have shown strong promise in coral reef restoration. Brink is theoretically tunable for different microbial communities and cell types, owing to the hydrogel scaffold's capacity to be optimized for key parameters such as gas exchange, molecular diffusion, and mechanical stiffness. As such, we envision Brink as a versatile technology platform that can be customized to support a range of settlement-inducing bacteria tailored to specific coral reef environments worldwide.

While the specific increase in larval recruit survival in situ is a focus of future work, even a 5-fold increase, if achieved, would be a promising advancement in leveraging sexual reproduction for reef restoration. Preliminary material cost estimates are about $25/L of prepolymer at scale. Brink polymers, applied in a thin layer (∼100 µm) at $0.25 L m^−2^, incurs a cost of $6.25 m^−2^. Considering noncontinuous application, with a maximum in crevices (10% coverage), the cost is about $0.63 m^−2^ for polymer materials. Additional expenses primarily relate to stock bacterial cultivation and coating applications, that could be undertaken by local nongovernmental organizations and communities. Importantly, application of this coating is timesaving as it avoids the long conditioning time usually required. While evident cost savings exist at an industrial scale, bulk part calculations suggest potential benefits could outweigh added costs. Increasing larval recruitment directly on substrates in the field would be a promising advancement in reef restoration, for both naturally occurring and reared coral larvae. That said, this technology is at an early stage as we have now shown the proof-of-concept (TRL 3). Important next steps include validation of this approach in situ and assessing longer-term benefits on coral recruitment (6–12 months).

Furthermore, light-assisted crosslinking is among the fastest methods for biomaterial processing, supporting its scalability. Future applications of Brink could involve direct 3D bioprinting onto substrates using light-assisted printing or batch polymerization with large-scale UV light sources (30). Additionally, Brink could be developed as a stand-alone settlement substrate with customizable architectures, further expanding its role in reef restoration efforts. Future work should refine hydrogel formulations to optimize bacterial persistence, cue diffusion, settlement efficacy and long-term survivorship under dynamic marine conditions, interactions with other organisms that could impact coral recruitment, and its efficacy with other coral species.

## Conclusion

In this study, we developed Brink, a bacterial reef ink that significantly enhanced coral settlement in laboratory experiments, increasing settlement of *M. capitata* and *P. damicornis* larvae by up to 5-fold compared with control substrates. The successful immobilization of bacteria within the hydrogel matrix suggests that Brink could remain stable for in situ applications. For large-scale applications in reef restoration and engineering projects, bacterial cultivation, material synthesis, and application of Brink must be done at scale. If successful, this microbial living material could be used to create effective settlement-inducing coatings for hybrid or biomimetic reefs (55), or 3D-printed settlement substrates (56, 57). Integrating Brink into reef restoration frameworks could provide a scalable and targeted solution to accelerate global coral restocking efforts.

## Materials and methods

### Bacterial strains and culture

Individual strains of the gram-negative bacteria *C. lytica* (HI1) isolated from biofilms from a seawater table at Kewalo Marine Laboratory, Honolulu, Hawai’i (39, 40) and *T. euphylliae* (H1) collected from *M. capitata* coral tissues (26, 38) were obtained from the Kewalo Marine Laboratory (University of Hawai’i, Oahu, Hawai’i). The two bacterial strains were streaked from −80 ℃ glycerol stocks onto 1/2 filtered seawater (FSW) tryptone (1/2 FSWt) agar plates and placed into a 25 ℃ incubator for 24–48 h and kept in the dark (23, 24). After incubation, 5 mL liquid bacterial cultures were prepared from 1/2 FSWt media and incubated for an additional 24 h at 25 ℃ on a shaker at 170 rpm. The liquid bacterial cultures were gently spun down (5,900 rpm, 30 min, 4 °C) to collect bacterial pellets that were resuspended in 10 mL of sterilized FSW to achieve an optical density (OD) of 1.00 at OD_600_ with a cell density of ∼10^8^ cells mL^−1^ for both strains.

### Living bacterial reef ink (Brink) fabrication

To develop a suitable living bacteria-loaded ink, we tested and optimized a variety of photocrosslinkable biopolymers. Biopolymers were chosen based on their known cell viability, mechanical stiffness, degradation properties, and ability to produce large batches for scalability. The combination of PEGDA and GelMA was chosen for their complementary mechanical properties (45). A combination of a higher percentage of PEGDA and a lower percentage of GelMA provides high resistance and minimal degradation of the hydrogel, which is a consequence of denser network structure providing higher crosslinking densities (58, 59).

GelMA was synthesized through a direct reaction of porcine gelatin (Sigma Aldrich, St Louis, MO, USA) and methacrylic anhydride (Sigma Aldrich) as reported previously (32, 33). PEGDA (Mn = 700 Da, Millipore-Sigma, St Louis, MO, USA) and the photoinitiator lithium phenyl-2,4,6 trimethylbenzoylphosphinate (LAP) (TCI America) were purchased. GelMA stock solutions (w/v) of 15 and 22.5% were prepared by dissolving lyophilized GelMA in MilliQ water. Brink was created by PEGDA, GelMA, LAP, and bacterial solution at a final concentration of 10% PEGDA, 7.5% GelMA, 0.5% LAP, and 10^8^ cells mL^−1^ of bacteria (*C. lytica* or *T. euphylliae*). The bioink was protected from light and kept at room temperature until polymerization.

### Rapid free radical photopolymerization of Brink

Calcium carbonate coral plugs (Ocean Wonders) were chosen as the substrate for the hydrogels due to its composition, porosity, neutral pH, texture, and rugosity that makes it highly compatible with corals and their larvae. The plugs were dried in a 65 ℃ oven, sanded, engraved on top with 1 mm crevices (Fig. 1), rinsed, and dried again before use. One hundred and twenty microliters of Brink were polymerized onto the plugs, yielding a roughly 500 μm thick microbial coating. Photopolymerization was induced via a UV light source (405 nm, Thorlabs, NJ, USA) at an irradiance intensity of 17 mW cm^−2^, which was optimized to facilitate successful crosslinking while minimizing potential cell damage (33).

### Larval settlement assays

We performed settlement assays with a broadcast spawning coral species (*M. capitata*) and a brooding species (*P. acuta*) from O’ahu, Hawai’i. For *P. acuta*, larvae were collected directly from adult colonies and introduced to settlement trials. *Montipora capitata* embryos were fertilized and reared to 96 h postfertilization following established best practices, before settlement experiments (Supplemental Materials).

Settlement assays were performed in 6-well plates containing 7 mL of FSW per well. We tested the efficacy of *C. lytica* Brink, *T. euphylliae* Brink, and uncoated control plugs. For all experiments, plugs were placed in 6-well plates upside down, with the top of the plug containing crevices face down in the well (*n* = 6 per treatment). Plugs were acclimated for a few hours in the well plates before adding coral larvae. Coral larvae were rinsed with FSW and counted to obtain an estimated number of larvae per milliliter. As a result of fewer numbers of colonies producing brooding coral larvae, the lower collection densities, and the larger size of the larvae (>2 times the size), settlement experiments that involved *P. acuta* (∼500 µm–1 mm larval length) used a density of ∼10–12 larvae per well for each treatment. Experiments with *M. capitata* (∼200–300 µm larval length) used 3 mL of larvae in FSW per well (∼40–45 per well), with a density of 15 larvae per milliliter for each treatment. Larvae were added to the wells and covered loosely with aluminum foil to facilitate oxygenation while preventing light exposure. Well plates were maintained overnight in a 27 ℃ incubator with a 12- to 12-h light–dark cycle at HIMB. After 12–15 h, plugs were agitated to remove nonattached larvae and the number of attached, dead/disintegrated, and swimming larvae in each well was quantified using microscopy via a stereo microscope (Motic SMZ-168) with a camera attachment (Cannon EOS 600D) (60).

### Microbial viability of Brink hydrogels

We tested bacterial growth and cell viability using *C. lytica* as our model strain, by coating common calcium carbonate–based restoration substrates with GelMA, PEGDA, and Brink hydrogels (PEGDA–GelMA; about 500 µm thick) and cultivated them for 1 week (Fig. 2B and C). Viability of *C. lytica* in the different polymers was assessed using the LIVE/DEAD BacLight Bacterial Viability Kit (Invitrogen, Thermo Fisher Scientific, OR, USA) and confocal microscopy. The kit was used following the manufacturer's protocol with minor modifications that included 15 min incubation with the stain at room temperature in total darkness followed by rinsing the hydrogel gently with sterile FSW. The stained hydrogels were then visualized using an inverted confocal microscope AxioObserver Z1 with LSM800 and Zen 2.6 blue edition software (Carl Zeiss, Oberkochen, Germany), a 20× objective lens (Plan-Apochromat 20×/0.8 M27), and optimized settings for EGFP (to visualize live bacteria; excitation 480/500 nm at 0.1–0.15% laser power, emission 488 nm, detection 410–546 nm), and TuRFP (to visualize dead bacteria; excitation 490/635 nm at 0.45% laser power, emission 561 nm, detection 400–650 nm). The Z-stacks (30 mm) were then presented as maximum intensity projection. Cell counts of the Brink coating were analyzed from confocal images in ImageJ2 Fiji (v.2.9.0/1.53t). Living and dead cells were quantified by creating a 16-bit black-and-white image. As the bacteria were rod shaped, the cells were then rounded, the background noise was reduced, the image was converted to binary, and the cells were then individually separated. Cell sizes were determined by pixel units (100–1500), outlined for circularity, and edges were excluded.

A calibration curve for reflectance was created by correlating the OD of bacterial liquid cultures with the absorbance (*De*) in Brink, to estimate bacterial density. OD was first measured in 2 mL of concentrated bacterial culture, followed by a serial dilution, where 1 mL of the culture was replaced with fresh media. OD was measured after each dilution, and this process was repeated eight times. The removed bacterial culture from each step was used to polymerize the living Brink coating, following the photopolymerization method previously described, and *De* was calculated for each dilution. OD in the liquid bacterial cultures was determined using a miniature spectrometer (Flame, Ocean Optics, USA; *n*  *=* 5 scans per measurement, boxcar width = 2 nm, resolution = 0.2 nm) in transmission mode. The liquid bacterial culture was placed into 3 mL cuvettes, and the absorption spectra were measured between 400 and 750 nm. Attenuation values were corrected by subtracting absorbance at 750 nm.

*De* spectra from Brink coatings were calculated from reflectance (*R*) measurements as *De* = Log(1/*R*) according to (61–63). Measurements were performed between 400 and 750 nm using a reflectance probe attached to a miniature spectrometer (Flame, Ocean Insight; *n*  *=* 5 scans per measurement, boxcar width = 2 nm, resolution = 0.2 nm). The probe was placed 5 mm away from the Brink coatings' surface at a 45° angle relative to the surface. Three random surface regions per bacterial coating were chosen for the measurements, and the experimental outcomes were normalized against a 99% diffuse reflectance standard (Spectralon, Labsphere, USA). Finally, bacterial growth in the Brink coating was monitored by determining bacterial *De* every day after polymerization for an entire week at 0 h, 12 h, 24 h, 48 h, 5 days, and 8 days after encapsulation of the bacteria. Samples (1 mL) for each isolate were fixed with 0.2 µm filtered formaldehyde (2% final concentration) for 15 min and counts were performed using a guava EasyCyte 5HT flow cytometer (Millipore©, USA).

### Mechanical stiffness

Mechanical properties were measured with a commercial device MicroSquisher (CellScale). Hydrogel cylinders of GelMA 22.5%, PEGDA 30%, and PEGDA–GelMA 30 and 15% with a height of 1 mm and a diameter of 1 mm, were printed using a custom-made 3D bioprinter (*n*  *=* 3). The hydrogel scaffolds were printed with a cylindrical mask (1 mm × 1 mm) onto methacrylate glass coverslips (405 nm for 30 s at 17 mW cm^−2^), using PDMS spacers with a thickness of 1 mm. Samples were compressed by a cantilever at the speed of 3 μm s^−1^ to reach the strain rate of 18%, and were held for 2 s, then recovered to the original size at a speed of 6 μm s^−1^. Each sample was compressed three times. The first two compression cycles removed hysteresis caused by internal friction. Young's elastic modulus was calculated using an in-house MATLAB code with the force and displacement data of the third compression cycle from the CellScale software.

### Microbial community of Brink coatings in seawater

To understand the potential changes to the monoculture bacterium community (i.e. *C. lytica*) inside the hydrogel environment when exposed to natural seawater, an ex situ aquarium experiment was performed at Scripps Institution of Oceanography (SIO). The aquarium setup contained a heater set at 26 °C, an air stone for oxygen, a powerhead for flow, and lighting set to a 12–12 h cycle (100 µmol m^−2^ s^−1^) to mimic natural conditions in a shallow reef ecosystem. Coral fragment plugs (Ocean Wonders) were sanded, rinsed, and evenly coated with a top layer (∼2 cm in diameter with a ∼500 µm thickness) containing either a Brink hydrogel or a control hydrogel (hydrogel containing no bacteria). On day 1 plugs with the Brink hydrogel (*n*  *=* 7) and control hydrogel (i.e. PEGDA–GelMA; *n*  *=* 7) coatings were polymerized with UV light and prepared according to the same protocol as described above. One plug from each treatment was immediately sampled by scraping the polymerized coating off the plug with a sterile scalpel blade and deposited into a 2-mL cryovial tube, which was immediately snap frozen at −80 °C prior to DNA extractions and 16S rRNA gene sequencing. Ambient 26 °C seawater was filtered (0.45 µm) from the SIO pier to obtain a seawater community similar to remove any particles and potential grazers. Both plug treatments were spaced and distributed in an egg-crate sitting mid-level in the same aquarium (at a level of ∼90 µmol m^−2^ s^−1^). A 1-L seawater sample was collected from the aquarium before plugs were placed, which was then vacuum filtered onto a 0.2-µm filters (Pall Supor 0.2 µm and 47 mm). After filtering the seawater three times, it was deposited into a cryovial tube and immediately snap frozen at −80 °C to preserve for further downstream analysis. Three plugs from each treatment were sampled again on day 3 (D3) and day 7 (D7). A 1-L seawater sample was collected on D3 before plugs were removed from the aquarium and filtered and preserved. Aquarium water was changed after the D3 plugs were removed and the D3 seawater samples were taken. New 26 °C FSW (0.45 µm) replaced the old water and a 1-L sample of the new seawater was collected, filtered, and preserved. The same seawater filtration and preservation process was repeated on D7 to mark the final bacteria community in the seawater at the end of the experiment.

DNA samples were extracted from Brink-coated coral fragment plugs, control hydrogel-coated plugs, and the seawater from the aquarium using the DNeasy PowerBiofilm Kit (Qiagen, Germany) according to the manufacturer's protocol. PCR amplification was conducted to target the 16S rRNA gene V4-V5 region (450 bp fragment length), using the universal primer set 515F and 907R (GTGCCAGCMGCCGCGGTAA and CCGTCAATTCCTTTGAGTTT, respectively) (64, 65). PCRs, amplicon purification, library preparation, amplicon sequencing, quality control, and bioinformatics were conducted by Novogene.

Raw reads were spliced and filtered to obtain clean data before using DADA2 to reduce noise (66). The remaining amplicon sequence variants (ASVs) were annotated to identify the related species information and abundance distribution (66). Reads were trimmed, filtered, denoized, merged, and chimeras were removed using the DADA2 workflow with QIIME2's classify-sklearn algorithm (67, 68), which is a Naïve Bayes classifier for species annotation of individual ASVs (trimmed to V4-5 region) using the 16S database SILVA v138.1 (69). The final ASVs were assigned to taxonomies and transferred from QIIME2 to R for statistical analyses.

### Statistical analyses

A one-way ANOVA and post hoc tests were performed for mechanical stiffness (compressive elastic modulus) of each biopolymer hydrogel scaffold. Proportion data for live and dead cells in different polymers and larval settlement assays were tested for normality of variance with a Shapiro–Wilk test and homogeneity of variance with Levene's test, where they failed to meet these assumptions (15). The data was log-transformed, which did not affect the normality or homogeneity of variance, and a nonparametric analysis was conducted with the Kruskal–Wallis rank sum test and the Dunn's post hoc test (15). Statistical significance was determined with *P* < 0.05 and bar graphs were plotted as averages with ±SD. ASV counts from QIIME2 and metadata were imported into R to conduct 16S amplicon bacteria community analyses, producing a heat map visualized via the pheatmap R package (70). All statistical analyses and plots were conducted using R (v4.0.3 and v4.2.1) (70).

## Supplementary Material

pgaf268_Supplementary_Data

## Data Availability

Data generated from this study is deposited on Figshare (10.6084/m9.figshare.29525534). Sequence data generated from this study has been deposited in the NCBI database under accession code PRJNA1289906.

## R3D Consortium

Ben Jones, Josh Levy, Sean Mahaffey, Aricia Argyris, Mark Aruda, Ian Robertson

Applied Research Laboratory at the University of Hawai’i

Zhenhua Huang, Ayrton Medina-Rodriguez, Mert Gokdepe, Brady Halvorson, Jon Chase, Charlotte White, Cami Dillon, Kristian McDonald, Anna Mikkelsen

University of Hawai’i at Manoa

Josh Madin, Mollie Asbury, Jessica Reichert, Hendrikje Jorissen, Nina Schiettekatte, Marion Chapeau, Rob Toonen, Chris R. Suchocki, Van Wishingrad, Christopher P. Jury, Dan Schar, Madeleine Hardt, Claire Lewis, Claire Bardin, Joshua Kualani, Crawford Drury, Kira Hughes, Josh Hancock, Carlo Caruso

Hawai’i Institute of Marine Biology

Andrea Grottoli, Shannon Dixon, Ann Marie Hulver

Ohio State University

Joshua D. Voss, Allison Klein, Siddhartha Verma, Alejandro Alvaro

Florida Atlantic University

Richard Argall, Kevin Chun, William Hicks, Alex LeBon, John Yeh

Makai Ocean Engineering

Aaron Thode, Océane Boulais, Daniel Wangpraseurt, Samapti Kundu, Natalie Levy, Stefan Kolle, Lindsey Badder

Scripps Institution of Oceanography, UC San Diego
